# Polypyrimidine tract binding protein 1 (PTBP1) contains a novel regulatory sequence, the rBH3, that binds the prosurvival protein MCL1

**DOI:** 10.1016/j.jbc.2023.104778

**Published:** 2023-05-03

**Authors:** Christine Carico, Jia Cui, Alexus Acton, William J. Placzek

**Affiliations:** Department of Biochemistry and Molecular Genetics, The University of Alabama at Birmingham, Birmingham, Alabama, USA

**Keywords:** BCL2 family, myeloid cell leukemia-1, NMR, polypyrimidine tract binding protein 1, RNA-binding protein, RNA, protein motif, ribonuclear protein, RNA processing, RNA recognition motif

## Abstract

The maturation of RNA from its nascent transcription to ultimate utilization (*e.g.*, translation, miR-mediated RNA silencing, etc.) involves an intricately coordinated series of biochemical reactions regulated by RNA-binding proteins (RBPs). Over the past several decades, there has been extensive effort to elucidate the biological factors that control specificity and selectivity of RNA target binding and downstream function. Polypyrimidine tract binding protein 1 (PTBP1) is an RBP that is involved in all steps of RNA maturation and serves as a key regulator of alternative splicing, and therefore, understanding its regulation is of critical biologic importance. While several mechanisms of RBP specificity have been proposed (*e.g.*, cell-specific expression of RBPs and secondary structure of target RNA), recently, protein–protein interactions with individual domains of RBPs have been suggested to be important determinants of downstream function. Here, we demonstrate a novel binding interaction between the first RNA recognition motif 1 (RRM1) of PTBP1 and the prosurvival protein myeloid cell leukemia-1 (MCL1). Using both *in silico* and *in vitro* analyses, we demonstrate that MCL1 binds a novel regulatory sequence on RRM1. NMR spectroscopy reveals that this interaction allosterically perturbs key residues in the RNA-binding interface of RRM1 and negatively impacts RRM1 association with target RNA. Furthermore, pulldown of MCL1 by endogenous PTBP1 verifies that these proteins interact in an endogenous cellular environment, establishing the biological relevance of this binding event. Overall, our findings suggest a novel mechanism of regulation of PTBP1 in which a protein–protein interaction with a single RRM can impact RNA association.

The controlled processing of RNA is a critical determinant in human development and cellular homeostasis, and its dysregulation has significant implication in human disease (1, 2, 3, 4, 5, 6). RNA processing (*i.e.*, 3′ and 5′ end processing, splicing, localization, stabilization, translation) is controlled by RNA-binding proteins (RBPs) and allows for adaptability of genomic information contained in a single gene to respond to cellular demands (7, 8, 9). Polypyrimidine tract binding protein 1 (PTBP1, also known as heterogeneous nuclear ribonucleoprotein I) is part of a class of RBPs known as heterogenous nuclear RBPs that are canonically associated with binding and nuclear processing (*i.e.*, splicing, polyadenylation) of nascent RNA as it is being actively transcribed (10, 11, 12). Accordingly, PTBP1 was initially described as a regulator of alternative splicing *via* its binding to pyrimidine-rich sequences near exon junctions (13, 14, 15, 16). However, continued biochemical interrogation into the functional roles of PTBP1 has demonstrated that it also has critical roles in most steps of RNA biogenesis (including but not limited to polyadenylation, mRNA stability, and internal ribosome entry site [IRES]–mediated translation) (17, 18, 19, 20, 21). Structurally, PTBP1 is a modular protein comprised of four RNA recognition motifs (RRMs) joined by three linker regions of variable length (22, 23). There are several types of protein domains capable of binding RNA (*e.g.*, zinc fingers and KH domains) (24), but the RRM domain is the most commonly occurring RNA-binding domain, underscoring its biological importance in RNA processing (25, 26, 27).

The canonical structure of the RRM domain is a β-sheet packed against two α-helices. The β-sheet forms the canonical RNA-binding interface of the RRM and contains conserved hexameric (RNP2 on the β1 strand) and octameric (RNP1 on the β3 strand) RNA-binding sequences (highlighted in Fig. 1*B*) (25, 26, 28). Prior structural analyses of RRM–RNA interactions by NMR and mass spectroscopy have demonstrated that only two to four of these conserved amino acid residues directly interact with the components of target single-stranded RNA (26, 29, 30, 31, 32, 33). Unsurprisingly, RNA sequence motifs bound by RBPs are very small and of low complexity (typically only a few nucleotides), and there is significant overlap with other RBPs (34). Specificity of RBP binding and downstream function is driven by several factors, such as the clustering of multiple RNA-binding domains in a single RBP, the nucleotide sequence surrounding the binding register (such as enhancer or silencer elements), secondary RNA structure, and protein–protein interactions with cofactors (33, 35, 36, 37).

Here, we identify a novel interaction between the first RRM of PTBP1 (RRM1) and myeloid cell leukemia-1 (MCL1) that is mediated by a 12-amino acid motif—termed the reverse B-cell homology domain 3 (rBH3) motif—in RRM1. MCL1 is a prosurvival member of the larger Bcl-2 family of apoptotic regulators and canonically antagonizes apoptosis at the outer mitochondrial membrane. The Bcl-2 family is functionally classified into two categories: proapoptotic (that are further subdivided into BH3-only proteins and effector proteins) and antiapoptotic proteins (such as MCL1) (38, 39, 40). The conserved structural unit of the Bcl-2 family of proteins is the BH3 motif, an amphipathic alpha helix that is present in all members of the Bcl-2 family (38, 39, 40). This helix is the signal transduction unit of the Bcl-2 family: if a BH3 helix binds the effector proteins BAX and BAK, these proteins oligomerize in the outer mitochondrial membrane, resulting in release of cytochrome *c* from the intermembrane space and subsequent caspase activation. If a BH3 helix is bound and sequestered by an antiapoptotic Bcl-2 family member (like MCL1), its proapoptotic effect is inhibited (38, 39, 40, 41). As we previously reported, the rBH3 sequence is a unique reversal of the canonical BH3 sequence that retains key conserved structural features (42) (Table 1). We previously demonstrated that the rBH3 motif is a functional regulatory sequence that allows MCL1 to modulate the function of rBH3-containing proteins. Specifically, we have demonstrated that MCL1 binds both the transcription factor p73 (a member of the p53 family of tumor suppressors) and the cell cycle regulator CDKN2C and inhibits their canonical functions in gene transcription and promotion of G1–S cell cycle progression, respectively (43, 44). Here, we demonstrate that MCL1 binds the RRM1 of PTBP1 *via* its rBH3 motif and propose a novel protein–protein interaction that can regulate RRM1 association with RNA.

**Table 1:** Comparison of conserved residues between the BH3 and rBH3 motif sequences

Key residues that drive MCL1 specificity are indicated in *red*. Hydrophobic residues that interact with hydrophobic pockets (p1–p4) within the BH3-binding groove of MCL1 are labeled H1–4. Both p18 and PTBP1 rBH3 sequences do not contain a hydrophobic residue at the H4 position.

## Results

### RRM1 of PTBP1 contains an rBH3 motif on its α2 helix

We recently established the rBH3 sequence as a naturally occurring and functional protein–protein interaction motif that allows MCL1 to bind to and regulate non-Bcl-2-family binding partners (*i.e.*, p73 and CDKN2C) (43, 44). Accordingly, we sought to identify additional cellular proteins that contain a putative rBH3 and are thus potential targets of MCL1 regulation. BLAST analysis of the human proteome identified a putative rBH3 in the first RRM1 of PTBP1 (Table 1). Within the MCL1 binding pocket, the canonical BH3 sequence makes several critical interactions: the conserved aspartic acid (D) of the BH3 sequence forms a salt bridge with a conserved Arg263 in the MCL1 binding pocket. In addition, four hydrophobic residues of the BH3 sequence (H1–4) insert into hydrophobic pockets within the MCL1-binding groove (p1–4) (Table 1) (38). The rBH3 sequence contains these conserved residues but in the reverse orientation so that they are positioned within conserved locations as compared with the BH3 sequence when read from C to N terminus (Table 1) (42). As observed in the rBH3-1 and rBH3-2 sequences, the PTBP1 rBH3 contains a homologous substitution in which the aspartic acid in the canonical BH3 sequence is replaced by glutamic acid (E105) (Table 1). An additional conserved substitution occurs at the H2 position, in which leucine in the canonical BH3 sequence is replaced by methionine (M110) (Table 1) (42). We have previously demonstrated that these substituted amino acids are critical for binding of other rBH3-containing proteins p73 and CDKN2C within the MCL1 binding pocket, as their mutation to alanine substantially reduced binding affinity by orders of magnitude (43, 44). In addition to M110 that is positioned to interact with the p2 pocket of the MCL1-binding groove, the PTBP1 rBH3 also contains hydrophobic residues at the H1 and H3 positions that are positioned to interact with respective hydrophobic pockets (p1, p3) within the MCL1-binding groove (Table 1).

**Figure 1:**
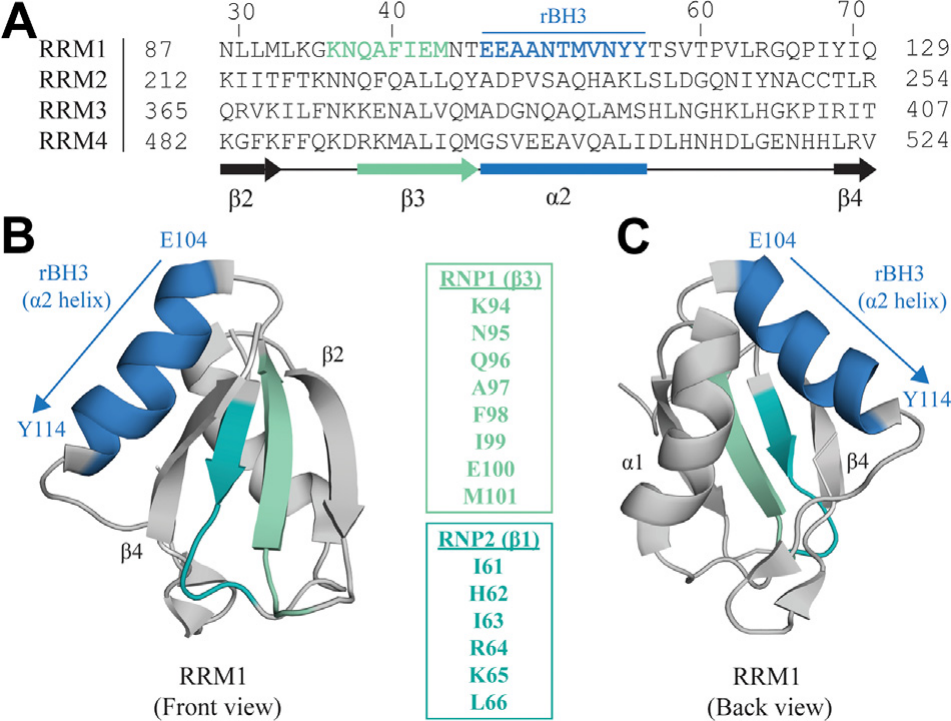
**Annotation of key structural features present in RNA recognition motif 1 (RRM1) of PTBP1**. *A*, MUSCLE protein sequence alignment of the four RRM1–4 of PTBP1 (UniProt ID: P26599). About 42 amino acids of each respective RRM are shown, residue numbers reflect those as reported in UniProt. Reverse B-cell homology domain 3 BH3 (rBH3) sequence is highlighted in *blue*. Secondary structure organization of RRM1 is shown below with *black arrows* referring to β strands, *gray boxes* referring to α helices, and *lines* referring to linker regions. *B* and *C*, front (*left*, *B*) and back (*right*, *C*) views of RRM1 (amino acid residues 58 through 133). rBH3 on the α2 helix is indicated, and N- and C-terminal residues are labeled (E104 and Y114, respectively; see Table 1). Secondary structure features are labeled, and key RNA recognition sequences, RNP1 on β3 (*green*) and RNP2 on β1 (*teal*), are indicated. This figure was generated with PyMOL (Protein Data Bank ID: 1SQW). PTBP1, polypyrimidine tract binding protein 1.

A key difference between BH3 and rBH3 sequences is that rBH3 sequences have thus far been identified in existing alpha helical regions of the protein, whereas BH3 helices in proapoptotic BH3-only proteins are intrinsically disordered until they associate with antiapoptotic proteins (39). Structural mapping of the rBH3 sequence of RRM1 revealed that, as was observed in p73 and CDKN2C, the rBH3 motif in RRM1 comprises the alpha2 helix (Fig. 1). Although BLAST sequence analysis identified a putative rBH3 sequence only in RRM1, the conserved topology of the RRM structure (αβ sandwich with a β1α1β2β3α2β4 topology (26)) led us to ask if there are any rBH3-like sequences in the other RRMs of PTBP1. MUSCLE (Multiple Sequence Comparison by Log-Expectation; https://drive5.com/muscle5/manual/citation.html) (45) sequence alignment of the four RRMs of PTBP1 (Fig. 1*A*) demonstrates that it is indeed only RRM1 that contains an rBH3 sequence, suggesting that MCL1 can interact with only RRM1 in an rBH3-dependent manner.

### RRM1 binds directly within the MCL1 binding pocket *via* an rBH3-mediated interaction

**Figure 2:**
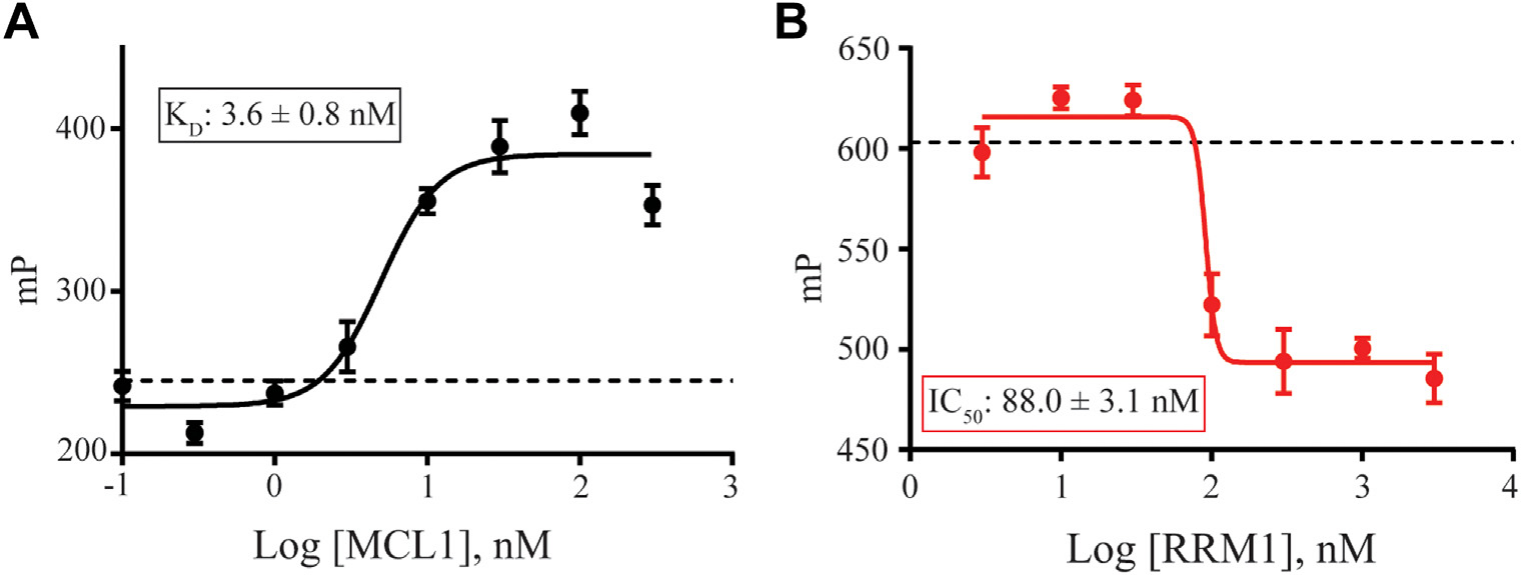
**RNA recognition motif 1 (RRM1) binds *via* its reverse B-cell homology domain 3 (rBH3) motif within the BH3 binding pocket of myeloid cell leukemia-1 (MCL1)**. *A*, direct fluorescence polarization anisotropy (FPA) consisting of 10 nM FITC-labeled RRM1_rBH3_ peptide (see Experimental procedures section for sequence) and a titration of recombinant unlabeled MCL1. *Dotted line* represents normalized average of a technical triplicate of 10 nM FITC-labeled RRM1_rBH3_ peptide alone (no MCL1 protein) control. *B*, competitive FPA data consisting of 100 nM recombinant MCL1, 10 nM FITC-labeled BAK (Table 1), and a titration of recombinant unlabeled RRM1. *Dotted line* represents normalized average of a technical triplicate of 10 nM FITC-BAK_BH3_ peptide with 100 nM recombinant MCL1 alone (no RRM1 protein) control. Lower millipolarization values indicate increased free fluorescent probe (*i.e.*, FITC-labeled RRM1_rBH3_ peptide).

We previously demonstrated through both fluorescence polarization (FP) and NMR data that the rBH3 motif of p73 and CDKN2C binds within the MCL1 BH3 binding pocket (43, 44). We thus hypothesize that RRM1 will also bind within the canonical BH3-binding groove of MCL1 *via* its rBH3 motif. We first confirmed that the rBH3 motif of RRM1 interacts directly with MCL1 utilizing a direct FP assay, as previously described (46). Briefly, 10 nM of a FITC-labeled peptide containing the RRM1 rBH3 sequence (F-RRM1_rBH3_) was incubated with escalating concentrations of recombinant MCL1 (1 nM–1 μM). We observed that F-RRM1_rBH3_ bound to MCL1 with a *K*_*D*_ of less than 10 nM, confirming that the rBH3 motif of RRM1 binds directly to MCL1 (Fig. 2*A*). Furthermore, we confirmed that the F-RRM1_rBH3_ peptide occupies the MCL1-binding groove, as pharmacologic inhibition of the MCL1-binding groove ablated binding (Fig. S1).

We next sought to confirm that the rBH3-containing full RRM1 protein binds within the MCL1 binding pocket. Such an interaction would displace the natural BAK BH3 sequence, an established ligand of the MCL1 BH3 binding pocket (Fig. S1) (43, 44, 47, 48). We thus utilized a competitive FP assay (46) in which 100 nM recombinant MCL1 was incubated with 10 nM of FITC-labeled BAK peptide derived from its BH3 sequence (F-BAK_BH3_) and escalating concentrations (1 nM–1 μM) of recombinant wildtype RRM1 protein. We observed that RRM1 was able to outcompete F-BAK_BH3_ from MCL1 as evidenced by a decrease in FITC-induced polarization (millipolarization) with increasing RRM1, with an IC_50_ of 88.0 ± 3.0 nM (Fig. 2*B*). As BAK is a well-characterized ligand of the MCL1 binding pocket, the ability of RRM1 to displace F-BAK_BH3_ confirms our hypothesis that this binding interaction occurs within the MCL1 binding pocket.

### MCL1 binding RRM1 displaces target RNA from RRM1

**Figure 3:**
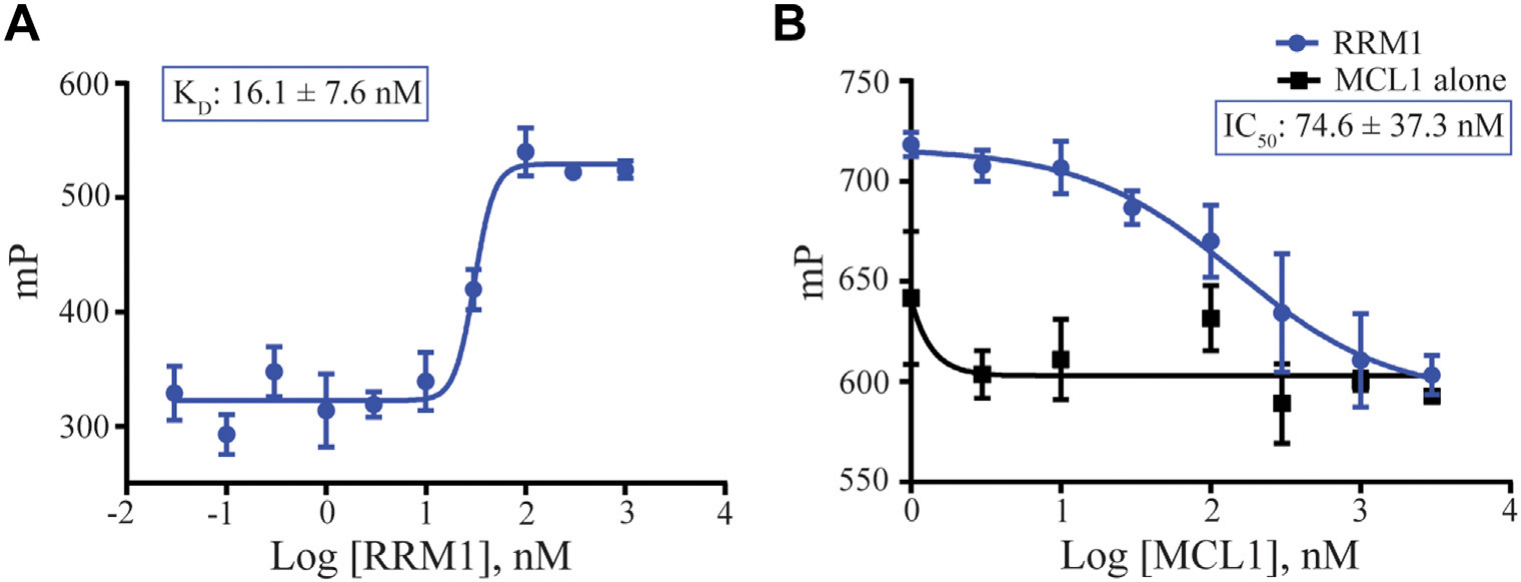
**Myeloid cell leukemia-1 (MCL1) disrupts RNA binding by RNA recognition motif 1 (RRM1).***A*, direct fluorescence polarization anisotropy (FPA) between RRM1 and 0.5 nM 25-mer FITC-labeled target RNA sequences found within the 3′UTR of the MCL1 mRNA (see Experimental procedures section for sequence). *B*, competitive FPA consisting of 50 nM RRM1, 0.5 nM FITC-labeled 25-mer of the 3′UTR of MCL1 mRNA, and a titration of recombinant MCL1 (*solid blue curve*). The *solid black line* refers to the normalized values of the MCL1 alone control. All FPAs were performed in a background of tRNA to reduce nonspecific binding. For both (*A* and *B*), lower millipolarization values indicate increased free fluorescent probe (FTSC-labeled RNA).

Our prior characterization of MCL1 binding with p73 demonstrated that MCL1 can negatively regulate p73 association with target DNA (43). Our FP data demonstrate that, like p73, RRM1 binds to the BH3 binding pocket of MCL1 *via* an rBH3-mediated mechanism. We therefore hypothesize that, similar to its impact on p73, MCL1 binding will impact RRM1 association with target RNA. Prior CLIP-Seq analysis revealed that PTBP1 binds to the 3′UTR of MCL1 (49), and we have shown that this enhances miR-101 targeting and subsequent AGO2-mediated degradation in the prostate cancer cell line PC3 (50). We therefore used a short mRNA derived from the 3′UTR of MCL1 to probe RRM1 RNA binding. It should be noted that this RNA probe was designed to act as an unstructured single-stranded RNA. As PTBP1 is a multi-RRM protein, we first utilized FP analysis to confirm that the RRM1 domain participates directly in binding to a known seed sequence in the 3′ UTR of MCL1 (Fig. 3*A*). To do this, we incubated increasing concentrations of RRM1 with 0.5 nM fluorescein-5-thiosemicarbazide (FTSC)–labeled 25-mer RNA derived from the 3′ UTR seed sequence of MCL1 and observed that recombinant RRM1 protein bound the target RNA with a *K*_*D*_ of 16.1 ± 7.6 nM as evidenced by the increase in millipolarization with increasing RRM1 concentrations.

After confirming RRM1 association with the target RNAs, we next asked if MCL1 can negatively regulate the association of RRM1 with this RNA. To test this, we utilized FP assay in which we incubated RRM1 with 0.5 nM FTSC-labeled 16-mer RNA and increasing concentrations of MCL1 (1 nM–1 μM). We observed that MCL1 displaces RNA from RRM1 with an IC_50_ of 74.6 ± 37.3 nM confirming that MCL1 can negatively regulate RRM1 association with target RNA (Fig. 3*B*). We confirmed this ability for MCL1 to displace RNA from RRM1 using the same FTSC–RNA in an EMSA-binding assay (Fig. S2).

### MCL1 binding RRM1 perturbs the conserved RNA-binding sequences, RNP1 and RNP2, within RRM1

**Figure 4:**
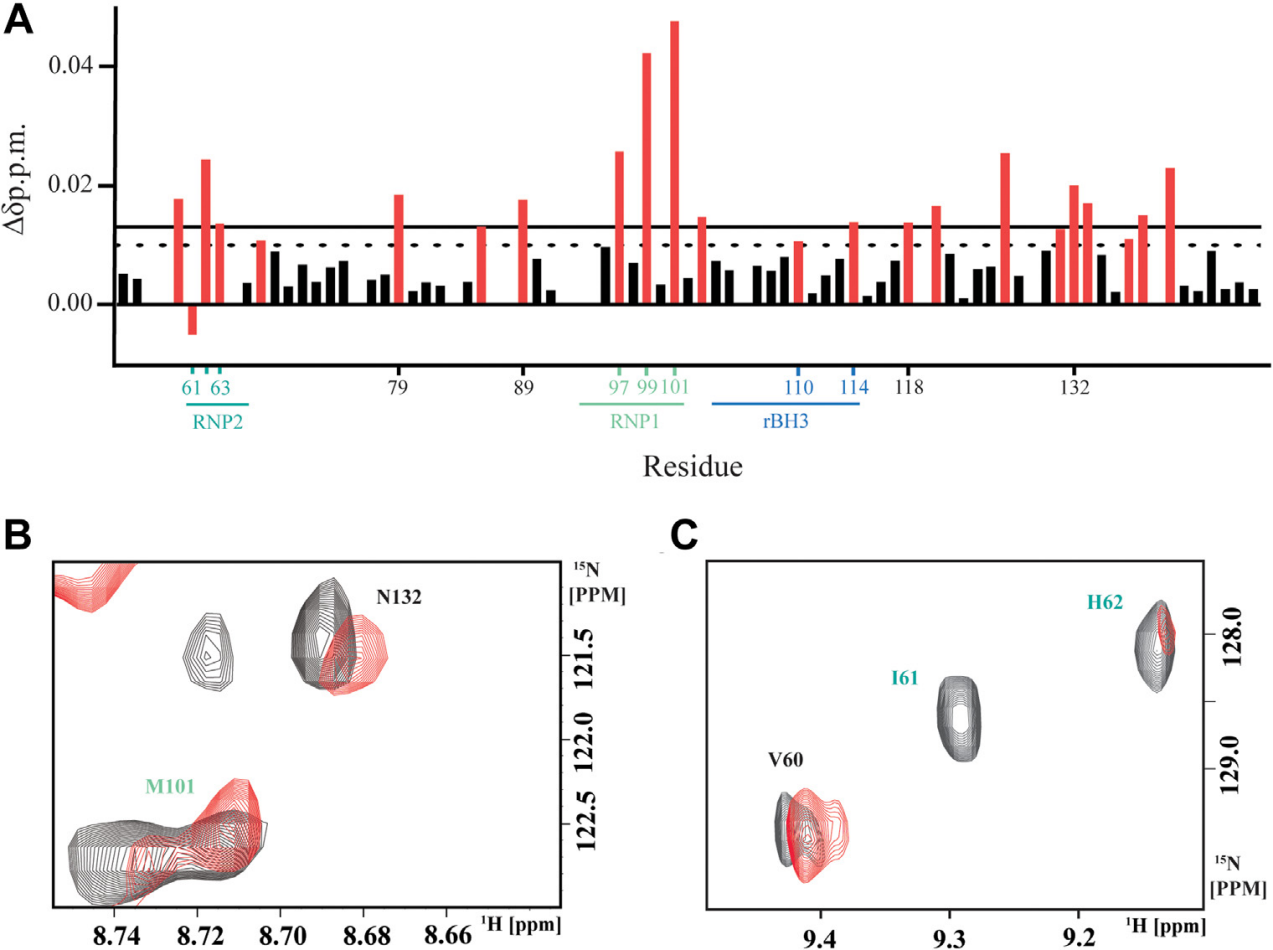
**Myeloid cell leukemia-1 (MCL1) binding RNA recognition motif 1 (RRM1) perturbs the conserved RNA binding sequences, RNP1 and RNP2**. *A*, chemical shift perturbations (CSPs) quantified as a function of Δδ ppm from 2D [^15^N, ^1^H]-heteronuclear single quantum coherence (HSQC) spectra of 20 μM ^15^N PTBP1–RRM1 as compared with 20 μM ^15^N PTBP1–RRM1 + 100 μM MCL1 from representative spectra. The *dotted line* indicates 1 SD from the mean (0.010), and the *solid line* indicates 2 SD from the mean (0.013). Amino acids with significant CSPs (defined as CSP 1 SD above mean: Δδ ≥0.010 ppm, *red bars*) were (V60, H62, I63, N69, G79, T86, L89, A97, I99, M101, T103, M110, Y114, T118, L121, Y127, S131, N132, H133, K137, T138, and S140). Select residues are indicated by number on the *x*-axis and are colored by secondary structure feature when appropriate (*i.e.*, rBH3, RNP1, RNP2—as seen for Fig. 1). Amino acids unable to be identified in the bound spectra were set to −0.005 to visually differentiate these residues from those with no CSPs (I61). Peaks unable to be identified in the apo protein were not plotted (S58, R59, R64, I76, K84, N87, L88, K92, G93, K94, A106, V120, Q129, and D139). *B* and *C*, expanded view of RNP1 and RNP2 from representative 2D [^15^N, ^1^H]-HSQC spectra of 20 μM ^15^N PTBP1–RRM1 (*black contours*) overlaid with 20 μM ^15^N PTBP1–RRM1 + 100 μM MCL1 (*red contours*). In (*B*) are shown residue M101 of RNP1 on the β3 strand as well as N132 on the β4 strand. In (*C*) are shown residues I61 and H62 of RNP1 as well as RNP2-adjacent V60 on the β1 strand. PTBP1, polypyrimidine tract binding protein 1; rBH3, reverse B-cell homology domain 3.

We sought to identify a mechanism by which MCL1 displaces RNA from RRM1, as MCL1 binds on the opposite side of the protein (the α2 helix) as the RNA-binding interface (the β-sheet) (Fig. 1, *B* and *C*). Given the organization of RRM1, we hypothesized that MCL1 binding the rBH3-containing α2 helix induces an allosteric perturbation of residues involved in RNA binding. To interrogate this, we employed NMR ^15^N chemical shift perturbation (CSP) mapping studies. ^15^N CSP analysis was chosen for these studies as the amide bond does an excellent job of reporting on changes in backbone chemical environment for all nonprolyl residues in a labeled protein, allowing us to conduct an unbiased analysis on the impact of MCL1 binding to RRM1. For these studies, we collected 2D [^1^H, ^15^N]-heteronuclear single quantum coherence NMR spectra of ^15^N labeled-RRM1 alone and combined with an excess of unlabeled recombinant MCL1 protein. All analyses were performed using three independent protein preparations of both RRM1 and MCL1 and collected on separate dates. Addition of MCL1 to ^15^N-RRM1 induced perturbation of the M110 residue at the H2 position of the rBH3 (Table 1 and Fig. 4, *A* and *C*) as well as terminal residues of the rBH3-containing α2 helix (Y114 in the H1 position [Table 1] and M101) (Fig. 4, *A* and *C*). Prior analysis of BH3 interactions with MCL1 has found that the H2 residue (M110 in PTBP1) forms a critical hydrophobic interaction with F270 in the p2 pocket of the MCL1-binding groove (47). As α2 is on the protein exterior and thus solvent exposed, we did not anticipate a large amount of backbone movement to orient the helix and critical residues to facilitate binding. Thus, we believe that perturbations in the terminal residues of the rBH3-containing α2 helix (M101 and Y114) reflect helical rotation of α2 to correctly orient the hydrophobic residues of the rBH3 for insertion into their respective hydrophobic pockets (p1–3) of the MCL1-binding groove.

Notably, we observed significant CSPs of residues located in both conserved RNA recognition sequences, RNP1 (I99, M101) and RNP2 (I61, H62, and RNP2-adjacent V60) (25, 26, 28) (Fig. 4). We also observed perturbation of S131, N132, and H133 that residue on the β4 strand that are important in forming the hydrophobic and hydrogen bond network that helps drive specificity of RNA sequence selection by RRM1 (29) (Fig. 4). Perturbation of residue H62 as well as residues S131, N132, and H133 are particularly significant as several structural studies of PTBP1’s RRM1 bound to RNA have demonstrated that these residues engage in pi-stacking with nitrogenous bases (H62) and stabilize the sugar backbone of single-stranded RNA (N132, H133) (29, 31). As our FP analysis functionally localizes the binding interaction between RRM1 and MCL1 to the rBH3 motif, we therefore consider CSPs of residues outside the rBH3 to be indicative of alternative biochemical events (*e.g.*, steric or allosteric events). These observed perturbations support a mechanism by which MCL1 binding the rBH3 motif on the alpha2 helix disrupts RNA binding by RRM1 as was observed in our FP and EMSA data (Figs. 3 and S2). Based on protein topology (*i.e.*, β3, α2, β4 are all immediately adjacent topologically), we hypothesize that these perturbations are due to allosteric mechanisms. However, without structural estimations of the complex, we cannot rule out the contribution of a steric event in which the larger MCL1 (∼21 kD) protein spatially engulfs some residues of the smaller RRM1 domain (∼12 kD), thus resulting in backbone perturbations observed outside the rBH3 motif.

### MCL1 associates with endogenous PTBP1 protein in a cellular environment

**Figure 5:**
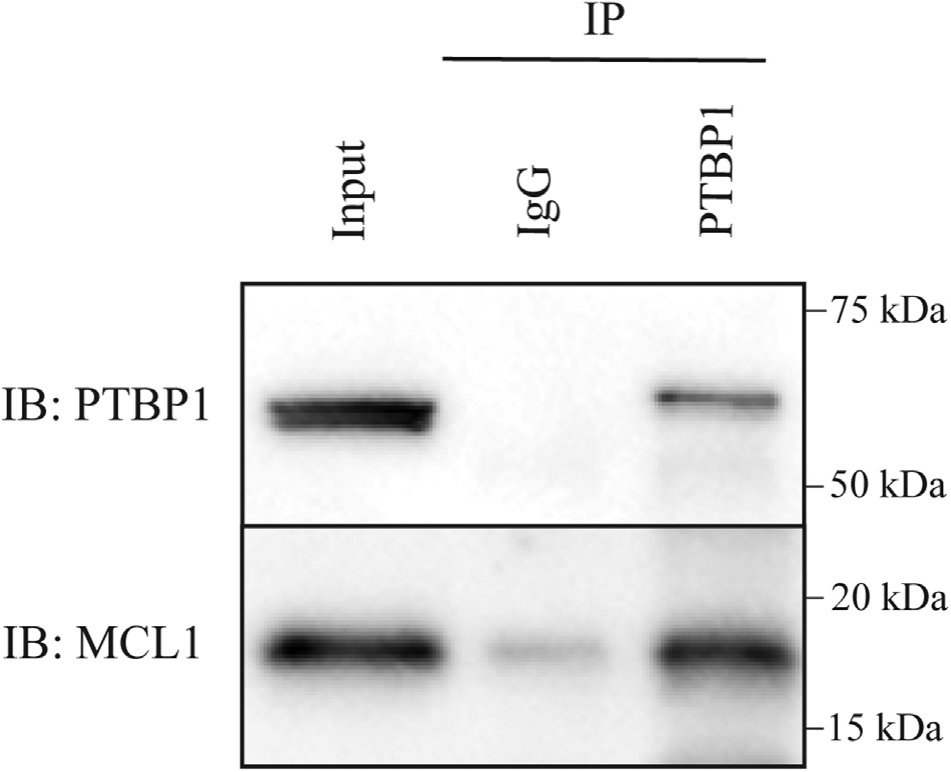
**Endogenous PTBP1 protein associates with myeloid cell leukemia-1 (MCL1) in a cellular environment.** Recombinant MCL1 was added to cellular lysate from the triple-negative breast cancer cell line MDA-MB-468. Pulldown was performed with anti-PTBP1 and captured by Protein G dynabeads. Western blot was used to confirm pulldown of MCL1 by PTBP1 (*versus* immunoglobulin G-nonspecific control). Lane markers on multichannel and colorimetric exposures for both PTBP1 and MCL1 immunoblots are visible in Fig. S3. PTBP1, polypyrimidine tract binding protein 1.

RBPs, including PTBP1, exist almost exclusively as part of larger multiprotein complexes bound to RNA transcripts at various stages throughout the RNA life cycle (11, 18, 24). The architecture of these larger protein complexes can spatially obstruct observed protein–protein interactions that have been established in an *in vitro* setting. While the focus of the current study is to biochemically define the rBH3-mediated interaction between RRM1 of PTBP1 and MCL1, as PTBP1 is often found in these larger protein complexes, we wanted to confirm the ability of PTBP1 to closely associate with MCL1 in an endogenous cellular environment (*i.e.*, against a background of competing protein–protein interactions). PTBP1 contains a bipartite nuclear localization and export signal at its N terminus and has been well established in prior literature to localize to both the nuclear and cytoplasmic compartments (51, 52, 53). While MCL1 is canonically considered a cytoplasmic protein, as this cellular subcompartment is where it exerts its antiapoptotic impact, we and others have previously demonstrated that MCL1 also has a biologically relevant localization in the nucleus and can interact with nuclear proteins (including, but not limited to, the rBH3-containing protein p73) (43, 54). We therefore hypothesize that MCL1 and PTBP1 can associate with one another in either cellular compartment and employed a pulldown of MCL1 by endogenous PTBP1 from whole-cell lysate. We observed that following immunocapture of endogenous PTBP1 by an anti-PTBP1 antibody (but not nonspecific control immunoglobulin G) with Protein G dynabeads, endogenous PTBP1 was able to selectively pull down recombinant MCL1 added to whole-cell lysate from the triple-negative breast cancer cell line MDA-MB-468. These data confirm that PTBP1 associates with MCL1 in a cellular environment and underscores the biological relevance of our biochemical data (Figs. 5 and S3).

## Discussion

**Figure 6:**
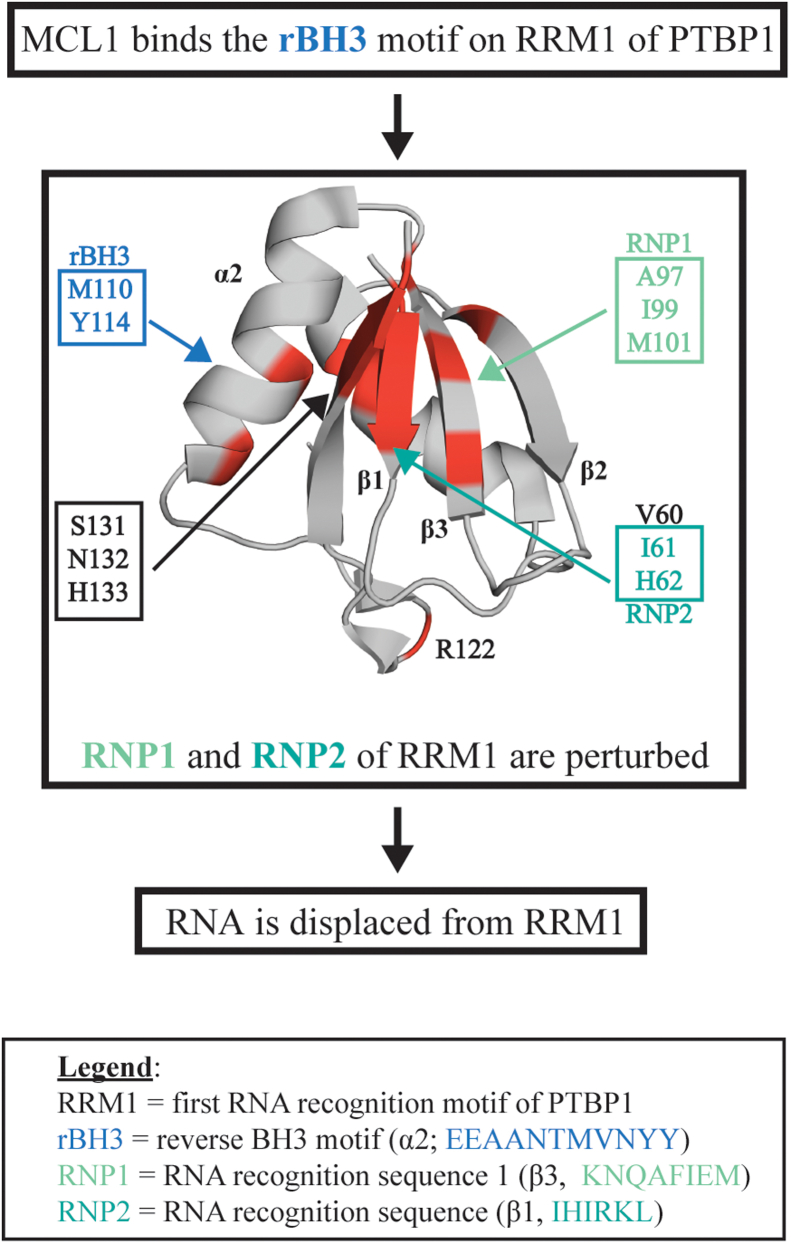
**Summary schematic: myeloid cell leukemia-1 (MCL1) binds the reverse B-cell homology domain 3 (rBH3) of RNA recognition motif 1 (RRM1) and perturbs RNP1 and RNP2, resulting in RNA displacement**. *Front cartoon* of RRM1 with residues that demonstrated either significant chemical shift perturbation (CSP) (Δδ ≥ 1 SD above the mean) or a shift in predominant conformation (*e.g.*, the emergence of a side peak of near equivalent intensity as the original main peak) repeated across biological triplicate spectra are colored in *red*.

In the current study, we report on the novel interaction between the first RRM of the RBP, PTBP1, and the apoptotic regulatory protein, MCL1. We have localized this interaction to the rBH3 motif on the α2 helix of the RRM1 domain of PTBP1 and to the BH3 pocket of MCL1. Our NMR data demonstrate that MCL1 binding to the rBH3 helix induces a conformational change in key residues involved in RNA binding (Fig. 6). Especially important are residues H62 on the β1 strand (that engages in pi-stacking with the nitrogenous bases of RNA) as well as N132 and H133 on the β4 strand (that form hydrogen bonds with the sugar backbone of single-stranded RNA) (29, 30). Prior NMR characterization of RNA-interacting residues of RRM1 has demonstrated that this H62 residue forms a key canonical pi-stacking interaction, and that N132 and H133 contribute hydrogen bonds that help define the sequence specificity of RRM1–RNA interactions, thus perturbation of these key RNA-interacting residues is particularly significant (26, 29, 30, 31, 32, 33). These studies are complemented by our FP data that demonstrate that MCL1 exhibits nanomolar binding to RRM1 that can displace a target RNA sequence (in this case, the 3′UTR of the MCL1 mRNA transcript). Importantly, this is only the second documented protein–protein interaction with a single domain of PTBP1 that has been shown to impact its function at target RNA. Likewise, NMR studies with a longer construct of PTBP1 (residues 41–163) have been used to capture additional C-terminal RRM1–RNA interactions, including formation of a C-terminal α-helix (comprised by residues 144–154), that folds upon binding RNA. The helix is partially formed in free RRM1 and becomes ordered upon binding to stem–loop encephalomyocarditis virus IRES RNA. This α3 helix does not directly interact with RNA and is instead thought to be a sensor of RNA secondary structure and act as an allosteric regulator of RNA binding (37). This suggests that RNA association with the RRM domain is highly regulated, and further analysis of protein regulators is justified.

The MCL1 binding pocket, into which the rBH3 of RRM1 binds, serves as the interface at which decisions regarding cell survival occur, as its canonical function is inhibiting apoptosis *via* binding and sequestering BH3 helices of proapoptotic Bcl-2 family proteins (38). We have recently shown that the MCL1 BH3-binding groove is conserved among all vertebrate classes, underscoring the evolutionary importance of this biological interface (55). Until recently, this apoptotic interface has historically been described as a terminal interaction within the apoptotic pathway, with no feed out to other cellular processes. However, our discovery of the rBH3 motif—a novel non-Bcl-2 family ligand of the MCL1 BH3 binding pocket—by our laboratories is establishing mechanisms that couple apoptotic signaling to other cellular processes. We have previously described the impact of MCL1 on gene transcription and cell cycle progression *via* regulation of rBH3-containing proteins p73 and CDKN2C, respectively (43, 44). The current study establishes a mechanism by which MCL1 can regulate association of RNA with RRM1 of PTBP1.

PTBP1 serves as a central regulator of gene expression—and by extension, cellular function—as it is intricately involved in all aspects of RNA maturation (17, 18, 19, 20, 21). As molecular techniques employed to study PTBP1 biology have matured, there is growing evidence that target-specific RNA processing events have RRM “dependencies,” or in other words, are driven by the activity of a single RRM of PTBP1 rather than a collective function of the entire protein. This begs the obvious question of whether there is RRM-specific regulation of function within PTBP1. Many protein-binding partners of PTBP1 have been described since its initial discovery in 1988; however, the RRM selectivity of these interactions is unclear, and accordingly, there are very few examples of domain-specific protein binding partners and regulators of PTBP1 function. Outside this work, the only other example of this is the interaction between RRM2 of PTBP1 and a 7-amino acid motif (SLLGEPP) on the Raver1 protein (56). Rideau *et al*. (56) have previously demonstrated that Raver1’s SLLGEPP-mediated interaction with RRM2 of PTBP1 suppresses the splicing of exon 3 of α-tropomyosin. This series of studies were the first to describe an RNA processing event as dependent on a single domain of PTBP1 (here RRM2) and to demonstrate that a protein–protein interaction can impact its domain-dependent function. Our study establishes a second peptide motif-mediated protein–protein interaction that, based on our identified mechanism, can impact the function of single RRM domain in PTBP1. It is entirely possible that MCL1 may also interact with the other RRMs of PTBP1 (by either nonspecific mechanism or *via* a currently undefined peptide motif) and thereby impact their specific function.

While to date there have been no transcriptome-wide analyses interrogating the role of RRM1 in overall RNA processing, previous targeted molecular studies have demonstrated the RRM1 dependency of RNA processing for several distinct targets. The RRM1 domain has been shown to be imperative for binding to and negative regulation of mRNA stability of both HIF-1α and AXL (a tyrosine kinase) transcripts as well as for alternative splicing of exon 10 of its homolog PTBP2 (57, 58, 59). While establishing the importance of RRM1 in the regulation of PTBP1 function at these targets, these studies did not provide a mechanism by which RRM1 could be regulated outside its deletion. Our current study provides such a mechanism for regulation of RRM1 association with RNA through a protein–protein interaction with MCL1 that allosterically perturbs key RNA-binding residues of RRM1. Our pull-down data suggest that this role is particularly important in disease states that rely on elevated MCL1 (*e.g.*, post–spinal cord injury, post–myocardial infarction, cancers) (60, 61, 62, 63). However, to understand how MCL1 impacts RRM1—and by extension, PTBP1—function, further analysis on the genome-wide role of RRM1 is needed. While the currently described domain-dependent functions of RRM1 are at regions of RNA that are unstructured and single stranded (*i.e.*, regions that undergo alternative splicing, miR targeting)—and thus, this was the RNA probe we selected for the current study—recent evidence suggests its importance in IRES-mediated translation. We therefore anticipate that RRM1 demonstrates domain-dependent function across all stages of RNA biogenesis and expect that such studies will provide needed insight into how MCL1-mediated cellular response to stress can move beyond the regulation of mitochondrial membrane integrity and into the regulation of cellular mRNA processing.

## Experimental procedures

### Protein sequence alignment

FASTA files for the protein sequence of each RRM1 of PTBP1 were downloaded from UniProt (P26599; RRM1: residues 59–143, RRM2: residues 184–206; RRM3: residues 337–411, and RRM4: residues 454–529). Sequence alignment was done using the MUSCLE (45) software program (version 3.8.31), and the resulting alignment file was visualized in Jalview (version 2.11.2.4; jalview.org).

### Recombinant protein purification

Human MCL1 (UniProt: Q07820; residues 163–326) and RRM1 (UniProt: P26599; residues 55–147) were cloned into a pET28a vector (EMD Millipore) to incorporate an N-terminal hexa-histidine tag (His6) and transformed into BL21 (DE3) *Escherichia coli* following the New England BioLabs protocol. About 1 l of bacterial cultures (4 × 250 ml/flask to allow for adequate aeration) were grown under kanamycin selection in Luria broth at 37 °C for 1.5 h. Using the cuvette reading on a Nanodrop 2000c Spectrophotometer, when an absorbance of 0.5 to 0.7 at 600 nm was reached, recombinant protein expression was induced using a final concentration of 1 mM IPTG (Fisher BioReagents). Cultures were grown for 4 h or until absorbance plateaued, and then harvested by centrifugation at 4700*g*. Cell pellet was subsequently frozen at −80 °C until further use. To lyse a bacterial pellet for protein purification, the pellet is resuspended in 20 ml protein lysis buffer (either 1× PBS [pH 6.8] or 1× Tris-buffered saline [TBS] + 2 mM β-mercaptoethanol [BME] [pH 6.8] for MCL1; 1× TBS + 1.5 M NaCl [pH 6.8] for RRM1) supplemented with two EDTA-free mini protease inhibitor tablets (Pierce; catalog no.: A32955) and 1× lysozyme (0.25 mg/ml) (Thermo Fisher). Resuspended pellet is then subjected to probe sonication for 6 to 8 min on ice before cellular debris is pelleted by centrifugation at 14,000*g* and filtered through a 0.45 μm syringe filter (Millex). Recombinant protein is then purified on a Bio-Rad NGC FPLC system using nickel chromatography (1 ml HisTrap; GE Healthcare) followed by gel filtration on a HiPrep 16/60 Sephacryl S-100 column (GE Healthcare). Fractions from both nickel chromatography and subsequent gel filtration were analyzed by SDS-PAGE to confirm the presence of protein of interest. MCL1 was stored in a final buffer of either 1× PBS (pH 6.8) (for direct fluorescence polarization anisotropy [FPA]) or 1× TBS + 2 mM BME (pH 6.8) (for competitive FPAs and NMR), and RRM1 construct was stored in a final buffer of 1× TBS + 2 mM BME (pH 6.8).

### Direct FPA

The FITC-RRM1_rBH3_ sequence used in Figure 2*A* is FITC-Ahx-NTEEAANTMVNYYTSVTPVLRGQ (GenScript). The FTSC-labeled RNA sequence (ACGCUUCUCUCAGGGAAAAACAUGC) used in Figure 3*A* was derived from the 3′UTR of the MCL1 mRNA transcript (synthesized by Integrated DNA Technologies). RNA was then 3′ end labeled with 5-FTSC.

In a flat-bottom and untreated black 96-well microplate (ThermoScientific), 90 μl recombinant protein is incubated with 10 μl 10× FITC-peptide or annealed FTSC–RNA (final concentrations of 10 nM and 0.5 nM, respectively). For the direct FPA between recombinant MCL1 and FITC-RRM1_rBH3_ (Fig. 2*A*), a seven-point titration curve was utilized with MCL1 concentrations ranging from 100 pM to 300 nM in half-log increments. For the direct FPA between recombinant RRM1 and FTSC–-RNA (Fig. 3*A*), a 10-point titration curve was utilized with RRM1 concentrations ranging from 30 pPM to 1 μM in half-log increments. The plate is then covered with an opaque lid and shaken at 300 rpm for 30 min before it is read using the FP-fluorescein setting (1.0 s, CW lamp filter—F485, emission filter—F535) on a PerkinElmer Victor X5 plate reader. All assays were performed in 1× PBS (pH 7.4) buffer with a final percent of dimethyl sulfoxide of 1%. Curve fitting was done using GraphPad’s Prism software using the equation Y = bottom + (top–bottom)/(1 + 10ˆ[(LogIC_50_-X) ∗ HillSlope]). All direct FP assays were performed in technical and biological triplicate using proteins purified from independent bacterial cell pellets on independent days. All data plotted in Figures 2 and 3 are one representative assay in technical triplicate.

### Competitive FPA

In a flat-bottom and untreated black 96-well microplate (ThermoScientific), 80 μl recombinant protein (100 nM final MCL1 in Figs. 2*B* and 50 nM final RRM1 in Fig. 3*B*) is incubated with 10 μl 10× unlabeled RRM1 (Fig. 2*B*, seven-point titration curve ranging from 300 pM to 3 μM in half-log steps) or MCL1 (Fig. 3*B*, eight-point titration curve ranging from 1 nM to 3 μM in half-log steps), and shaken at 300 rpm for 20 min to allow for binding. After the initial 20 min incubation, 10 μl of 10× FITC-BAK (Fig. 2*B*, 10 nM final concentration) (sequence: FITC-Ahx-GQVGRQLAIIGDDINRRYD) or FTSC–RNA (Fig. 3*B*, 0.5 nM final concentration) is added, and the plate is covered with an opaque lid and shaken for an additional 40 min at 300 rpm. Plate is then read using the FP–fluorescein setting (1.0 s, CW lamp filter—F485, emission filter—F535) on a PerkinElmer Victor X5 plate reader. All assays were performed in 1× PBS (pH 7.4) buffer with a final percent of dimethyl sulfoxide of 0.1% (Fig. 2*B*) or 1% (Fig. 3*B*). Curve fitting was done using GraphPad’s Prism software using the equation Y = bottom + (top–bottom)/(1 + 10ˆ[(LogIC50-X)∗HillSlope]). All competitive FP assays were performed in technical and biological triplicate using proteins purified from independent bacterial cell pellets on independent days. All data plotted in Figures 2 and 3 are one representative assay in technical triplicate.

### NMR

The NMR heteronuclear single quantum coherence spectra were acquired using a Bruker 600 MHz magnet at the Central Alabama High Field NMR Facility at the University of Alabama at Birmingham. Samples were freshly prepared on the day of collection in 1× TBS with 2 mM BME, pH 6.8, supplemented with sodium azide (Fisher BioReagents) and deuterium oxide (99%; Cambridge Isotope Laboratories, Inc). The collected spectra were analyzed using computer-aided resonance assignment, and the peak lists were exported into Microsoft Excel for subsequent analysis. Briefly, CSPs identified using a combination of nearest neighbor and comparison to previous analysis of rBH3 binding and were quantified by calculating the ΔΔppm of each amino acid residue using the formula: √Δδ_H_^2^ + (Δδ_N_/5)^2^. Mean and standard deviation of the CSP for each residue was calculated in Microsoft Excel, and any residues demonstrating CSP >1 SD were considered significant. All spectra were collected in biological triplicate, and residues that demonstrated significant CSP in all three spectra were mapped to the ribbon model of RRM1 on PyMOL (pymol.org), using Protein Data Bank file 1SJQ: NMR structure of RRM1 from human polypyrimidine tract binding protein isoform 1 (PTB1). In Figure 4, *A*–*C*, CSPs are plotted by residue, and examples of overlaid raw data, respectively, are shown from a representative spectra.

### Pulldown

MDA-MB-468 triple negative breast cancer cells were lysed in 1 ml of 1× immunoprecipitation lysis buffer (Pierce): 25 mM Tris–HCL (pH 7.4), 150 mM NaCl, 1 mM EDTA, 1% NP-40, and 5% glycerol in water supplemented with protease inhibitor (Halt Protease Inhibitor; ThermoScientific). Total protein amount was determined by bicinchoninic acid assay (ThermoScientific). Volume of cellular lysate corresponding to 1 mg of total protein was incubated with 25 μg recombinant MCL1 protein and 2.5 μg of either anti-PTBP1 primary antibody (RN011P,; MBL) or control immunoglobulin G antibody (DA1E; Cell Signaling) rotating overnight at 4 °C. The immunoprecipitation reaction was then pulled down with 25 μl protein A/G magnetic beads (Pierce) at room temperature for 2 h, washed 3× in 500 μl lysis buffer, and eluted. Pulldown of MCL1 by endogenous PTBP1 was assayed by Western blot to visualize both PTBP1 (MBL; primary antibody in 1:1000 dilution) and MCL1 (D35A5; Cell Signaling; primary antibody in 1:1000 dilution) and imaged on a Bio-Rad ChemiDoc MP imaging system.

### EMSA

About 30 μM recombinant RRM1 (purified in 1× TBS with 2 mM BME at a pH of 6.8) was incubated with 50 nM FTSC-labeled RNA derived from the 3′UTR region of the MCL1 mRNA transcript (see Direct FPA section of Experimental procedures for sequence) in a buffer containing 10 mM Hepes (pH 7.3), 20 mM KCl, 1 mM MgCl_2_, 1 mM DTT, 10 μg/ml tRNA (ThermoFisher; catalog no.: 20159) for 30 min at room temperature. Recombinant MCL1 (purified in 1× TBS with 2 mM BME at a pH of 6.8) was then added (to final concentrations of 50, 100, 140 μM) and incubated for an additional 40 min at room temperature. After addition of nucleic acid loading buffer (ThermoFisher; catalog no.: R0611), samples were loaded on a 1% Tris–borate–EDTA agarose gel and electrophoresed for 20 min at 120 V at room temperature. At all steps of the protocol, samples and gel were protected from light to prevent fluorophore quenching.

## Data availability

The individual RRM sequences of PTBP1 as well as the generated MUSCLE sequence alignment as visualized in Jalview are available in the supporting information files. NMR peak lists have been deposited in Biological Magnetic Resonance Bank (ID: 51712).

## Supporting information

This article contains supporting information.

## Conflict of interest

The authors declare that they have no conflicts of interest with the contents of this article.

## Supporting information

Supporting information

PTBP1_RRM_alignment

PTBP1_RRM_sequences
