# Supplementary material for: Polypyrimidine tract binding protein 1 (PTBP1) contains a novel regulatory sequence, the rBH3, that binds the prosurvival protein MCL1
Source: J Biol Chem. 2023 May 3;299(6):104778. doi: 10.1016/j.jbc.2023.104778 (PMC10244698; doi:10.1016/j.jbc.2023.104778)
Supplement: Supporting information [file mmc1.pdf]

Polypyrimidine tract binding protein 1 (PTBP1) contains a novel regulatory sequence, the rBH3, that binds the pro-survival protein MCL1

Christine Carico[1], Jia Cui[1], Alexus Acton[1], William J. Placzek[1*]

[1]Department of Biochemistry and Molecular Genetics, The University of Alabama at Birmingham, Birmingham, AL 35294

Material Included:

Figure S1

Figure S2

Figure S3

Separate Supplementary Data Files:

PTBP1_RRM_alignment.zip

- Jalview file of visualized MUSCLE alignment of all PTBP1 RRM sequences

PTBP1_RRM_sequences

- individual PTBP1 RRM sequences used for alignment

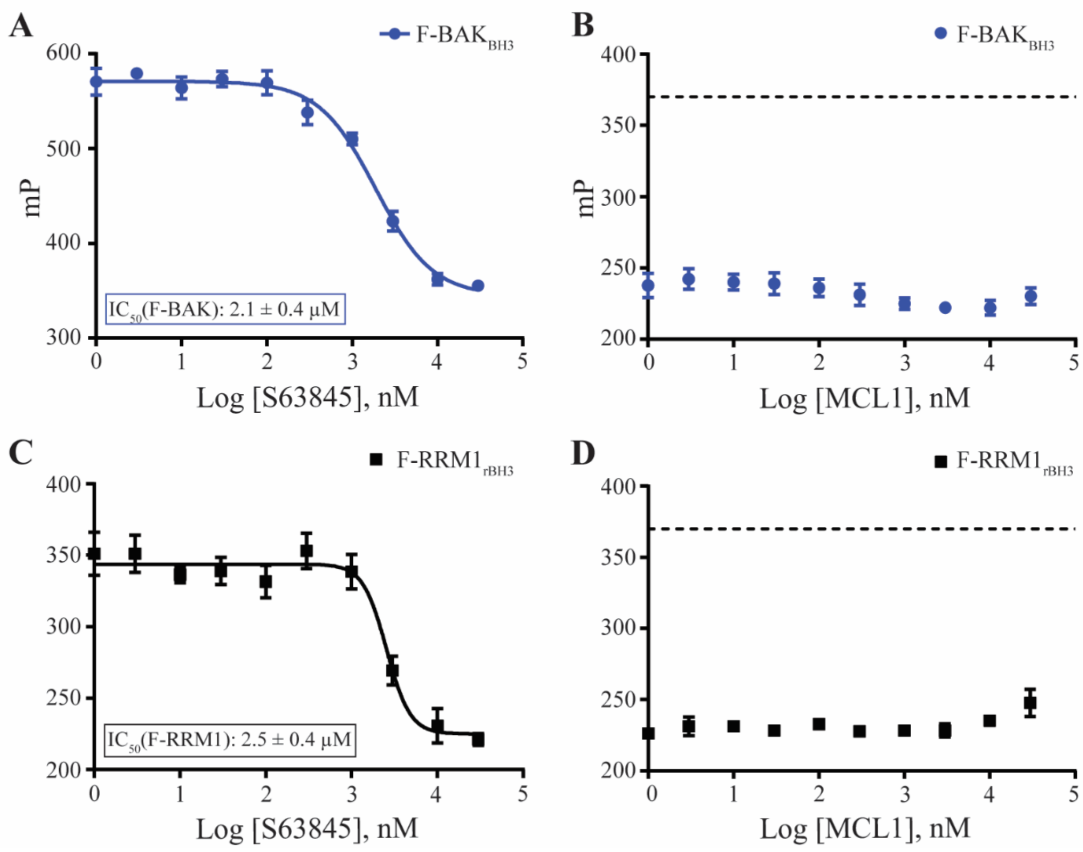

Figure S1. FITC-RRM1$_{rBH3}$ and FITC-BAK$_{BH3}$ peptides occupy the MCL1 binding pocket. A) and C) Competitive FPA consisting of 100 nM recombinant MCL1 protein, 10 nM of either FITC-labeled BAK$_{BH3}$ (A) [see Table 1 for sequence] or FITC-labeled RRM1$_{rBH3}$ peptide (C) [see Experimental Procedures for sequence] and a titration (1 nM-30 µM) of the selective MCL1 binding pocket inhibitor S63845. Lower mP values indicate more free fluorescent probe (i.e., FITC-labeled BAK$_{BH3}$ peptide and FITC-labeled RRM1$_{rBH3}$ peptide, respectively). B and D) Direct FPA consisting of 10 nM F-BAK$_{BH3}$ peptide (B) or FITC-labeled RRM1$_{rBH3}$ peptide (D) and a titration of MCL1 (10-points, 1 nM – 30 µM) in a background of 3 µM S63845. Dotted line represents the normalized value of 10 nM FITC- RRM1$_{rBH3}$ peptide alone in 1% DMSO.

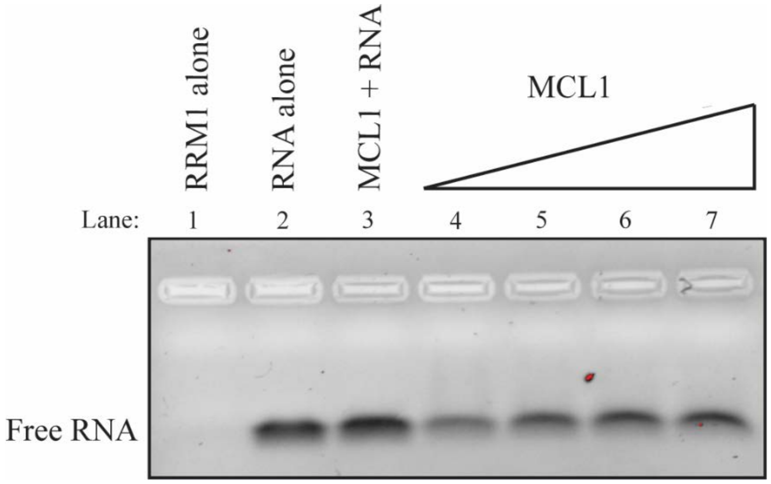

Figure S2. MCL1 displaces target RNA from RRM1. Electromobility shift assay (EMSA) consisting of 30 µM recombinant RRM1 protein (lanes 1, 4-7), 50 nM FTSC-labeled RNA (lanes 2-7) [derived from 3'UTR of MCL1 mRNA, see Figure 3 and Experimental Procedures]. MCL1 titration involved 4 concentrations of recombinant MCL1 protein: 0 µM (lane 4), 50 µM (lane 5), 100 µM (lane 6), 140 µM (lane 7). Assays were run against a background of tRNA (10 µg/µL) to reduce non-specific binding.

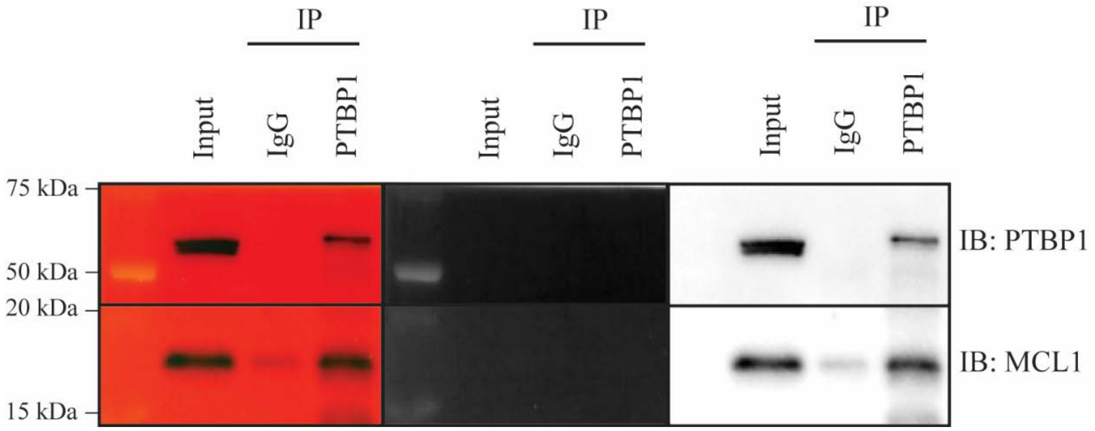

Figure S3. **Lane markers for MCL1 pulldown by endogenous PTBP1**. As in Figure 5, recombinant MCL1 was added to cellular lysate and pulled down by endogenous PTBP1 immunocaptured by anti-PTBP1 antibody on Dynabeads. All three exposures for both PTBP1 and MCL1 immunoblots are shown above (from left to right) in order to visualize lane markers above and below: multichannel, colorimetric, Chemi Hi sensitivity.
