# Supplementary material for: Polypyrimidine tract binding protein 1 (PTBP1) contains a novel regulatory sequence, the rBH3, that binds the prosurvival protein MCL1
Source: J Biol Chem. 2023 May 3;299(6):104778. doi: 10.1016/j.jbc.2023.104778 (PMC10244698; doi:10.1016/j.jbc.2023.104778)
Supplement: PTBP1_RRM_sequences [file mmc3.docx]

>sp|P26599|59-143

RVIHIRKLPIDVTEGEVISLGLPFGKVTNLLMLKGKNQAFIEMNTEEAANTMVNYYTSVT

PVLRGQPIYIQFSNHKELKTDSSPN

>sp|P26599|184-260

LRIIVENLFYPVTLDVLHQIFSKFGTVLKIITFTKNNQFQALLQYADPVSAQHAKLSLDG

QNIYNACCTLRIDFSKL

>sp|P26599|337-411

SVLLVSNLNPERVTPQSLFILFGVYGDVQRVKILFNKKENALVQMADGNQAQLAMSHLNG

HKLHGKPIRITLSKH

>sp|P26599|454-529

ATLHLSNIPPSVSEEDLKVLFSSNGGVVKGFKFFQKDRKMALIQMGSVEEAVQALIDLHN

HDLGENHHLRVSFSKS
